# Impact of a Sepsis Quality Improvement Initiative on Clinical and Operational Outcomes

**DOI:** 10.3390/healthcare13111273

**Published:** 2025-05-28

**Authors:** Christopher B. Thomas, Benjamin Wyler, Claude M. D’Antonio, Mark Laperouse, Shannon Alwood, Kristen Richard, Alyse Grantham, Roya Sheybani, Matt G. Sorrells, Wei-Jien Tan, James W. Teague, Hollis O’Neal, Tonya Jagneaux

**Affiliations:** 1 Department of Pulmonary and Critical Care, Our Lady of the Lake Regional Medical Center, Baton Rouge, LA 70808, USA; christopher.thomas@fmolhs.org (C.B.T.); tonya.jagneaux@fmolhs.org (T.J.); 2 Department of Pulmonary and Critical Care, LSU Health Sciences Center, Baton Rouge, LA 70808, USA; 3 Franciscan Missionaries of Our Lady Health System, Baton Rouge, LA 70809, USA; 4 Department of Emergency Medicine, Icahn School of Medicine at Mount Sinai, New York, NY 10029, USA; benjamin.wyler@mountsinai.org; 5 Department of Emergency Medicine, Our Lady of the Lake Regional Medical Center, Baton Rouge, LA 70808, USA; claude.dantonio@pepaem.com (C.M.D.); mark.laperouse@pepaem.com (M.L.); salwoo@lsuhsc.edu (S.A.); kristen.richard@fmolhs.org (K.R.); alyse.grantham@fmolhs.org (A.G.); 6 Department of Emergency Medicine, LSU Health Sciences Center, Baton Rouge, LA 70808, USA; 7 Cytovale Inc., San Francisco, CA 94080, USA; roya.sheybani@cytovale.com (R.S.); matt.sorrells@cytovale.com (M.G.S.); wei.tan@cytovale.com (W.-J.T.); 8 Core Laboratory & Blood Services, Our Lady of the Lake Regional Medical Center, Baton Rouge, LA 70808, USA; james.teague@fmolhs.org

**Keywords:** sepsis, host response, risk stratification, emergency department, quality improvement, length of stay, blood culture, healthcare utilization, antibiotic stewardship, deformability cytometry

## Abstract

**Background/Objectives:** Sepsis is a costly and life-threatening condition caused by a dysregulated host response to infection. Lack of a reliable, timely diagnostic for sepsis leads to under- and overdiagnosis, suboptimal outcomes, and strained hospital resources. Our Lady of the Lake Regional Medical Center (OLOLRMC) implemented a sepsis learning health program to evaluate and improve outcomes through standardized ED workflows and the incorporation of a novel sepsis diagnostic test. **Methods:** We report the results of the first year of experience following the implementation of the learning health initiative and sepsis testing. Data from the Epic EHR were analyzed across two groups: pre-implementation (April 2023–July 2023) vs. post-implementation (August 2023–July 2024), and temporally matched cohorts (April–July 2023 vs. April–July 2024). We assessed clinical outcomes (sepsis-associated mortality, hospital length of stay, or HLOS), and resource utilization (antibiotic use, blood cultures). **Results:** Post-implementation, sepsis-associated mortality dropped from 10.9% to 6.6% in the temporally matched group (*p* < 0.001). There was also a 0.76-day reduction in mean HLOS among sepsis DRG patients (*p* < 0.05). Blood culture utilization fell from 50.8% to 45.7%, driven by reductions in blood culture utilization among patients receiving a Band 1 IntelliSep score. **Conclusions:** The FMOLHS experience demonstrated significant benefits to patient outcomes and resource utilization after implementing a sepsis QI initiative including protocolized and standardized ED workflows via a nurse-driven triage system with sepsis testing for the early risk stratification of patients who present to the ED with signs and symptoms of infection.

## 1. Introduction

Sepsis is defined as life-threatening organ dysfunction caused by a dysregulated host response to infection [1]. It remains a leading cause of mortality [2,3,4] and a significant driver of healthcare costs [5]. Its heterogeneous presentation and historic lack of diagnostic aids makes sepsis one of the most common misdiagnoses in the emergency department (ED) [6], contributing to both inefficient care and suboptimal outcomes.

The effective application of sepsis clinical guidelines presents challenges because of variability and subjectivity in the diagnosis, even among experienced clinicians [7,8]. Additionally, current performance measures [9] may lead to inappropriate resource allocation (blood cultures, broad-spectrum antibiotics), diagnostic anchoring [10], and potential harm to patients who have other time-sensitive, life-threatening diseases.

On 1 August 2023, Our Lady of the Lake Regional Medical Center (OLOLRMC) became the first hospital to integrate a novel sepsis diagnostic, the IntelliSep test, into its comprehensive sepsis program. IntelliSep is a diagnostic aid that quantifies the structural changes that immune cells undergo upon activation [11,12]. IntelliSep’s three interpretation bands [13] allow for the operationalization of the surviving sepsis guidelines by providing three discrete clinical pathways of care: ‘Sepsis Unlikely’ (Band 1), ‘Sepsis Possible’ (Band 2), and ‘Sepsis Probable or Definite’ (Band 3).

In this manuscript, we report the impact of a Sepsis Learning Health Initiative featuring the IntelliSep test on sepsis outcomes and resource utilization, using data gathered between April 2023 and July 2024. The reported outcomes include patient-centered data (mortality, HLOS) and resource utilization (blood culture orders and antibiotic administration).

## 2. Materials and Methods

OLOLRMC is an 800-bed academic medical center in Baton Rouge, Louisiana. In July 2022, we developed a sepsis performance improvement (PI) program [14] within the Quality and Safety infrastructure. A summary of interventions is found in Section S1.

In April 2023, OLOLRMC implemented two electronic health record-enabled (EHR) sepsis screening tools via our practice advisories (OPA) process. This process includes two distinct OPA mechanisms: a primary (Triage) OPA and a secondary (Provider) OPA. The triage OPA is dependent upon the triage vitals and nursing questionnaire, while the provider OPA is dependent upon the Epic Sepsis Model, a predictive analytic model which reports a score every 20 min. Details of the Triage OPA and its associated logic are available in the Section S2.

In August 2023, the IntelliSep test was made available through these OPAs. Three discrete IntelliSep-informed clinical pathways were developed to help guide ED providers: Band 1 (‘Sepsis Unlikely’), Band 2 (‘Sepsis Possible’), and Band 3 (‘Sepsis Probable’). These pathways are based on the consensus guidelines of the Surviving Sepsis Campaign with specific recommendations informed by data collected from prior IntelliSep studies [15].

Providers were educated on these pathways during the first month of implementation through a series of journal clubs and ‘lunch-and-learn’ activities conducted within the first month of implementation. Following these initial educational activities, no formal education or feedback was provided to clinicians regarding ED interventions. Because the provider education occurred during the first month of IntelliSep implementation, August 2023 serves as a transition period. Following this initial training, clinicians received no formal education regarding these ED interventions. Between 1 August 2023 and 31 July 2024, there were no additional interventions or PI projects impacting the components of sepsis care (e.g., blood culture utilization/contamination, antimicrobial stewardship) in the hospital.

The local IRB (LSU Health Sciences Center Protocol #7501) determined the initiative to be quality improvement. This report includes adult (>18 years) patients who presented to the OLOLRMC ED from April 2023 through July 2024 and for whom either OPA was issued. Data were sourced from the EHR, then collated and maintained by the FMOLHS Quality and Safety department. Deidentified data were made available to Cytovale through a Data Use Agreement. Data analysis was conducted in collaboration with the hospital’s Quality and Safety team and Cytovale, with the Quality and Safety team leading the analysis and giving final approval for all results.

This analysis describes the impact of the intervention on patients for whom any sepsis screening OPA was activated, regardless of the ordering of the sepsis test. Patients for whom an OPA was activated between 23 April 2023 (when the OPA process was implemented) and 31 July 2023 serve as a pre-implementation ‘baseline’ analysis group. Patients for whom an OPA was activated between 1 August 2023, and 31 July 2024, will serve as the post-implementation group. Post-implementation data were split into two separate cohorts for analysis: the ‘post-implementation’ group (August 2023–July 2024) and, to mitigate the effect of seasonal variation, a ‘temporally matched’ group (April–July 2024).

Outcomes include in-hospital mortality and HLOS, while resource utilization includes ED blood culture and antimicrobial utilization. Baseline characteristics and descriptive statistics are presented as counts, percentages, means, standard deviations, medians, and first and third quartiles. HLOS calculations exclude patients who died in hospital or were discharged to a hospice, and the non-parametric Mann-Whitney U test is used for comparison. The chi-squared proportions test is used to test the differences in populations for binary and categorical data. Sepsis-associated mortality is defined as mortality occurring in patients whose a hospital stay was assigned a final diagnosis-related group (DRG) code of 870, 871, or 872. We compared the Charlson Comorbidity Index categories between the pre and post and the pre and matched cohorts by evaluating the proportion of patients meeting each category using the chi-square tests of proportions. To account for multiple hypothesis testing, *p*-values were adjusted using the Benjamini–Hochberg procedure to control the false discovery rate, which is appropriate given the multiple comparisons across 17 categories and the potential correlation between comorbidities. After adjustment, no categories remained statistically significant at a threshold of *p* < 0.05. Prior to correction, two categories, namely cerebrovascular disease and paraplegia/hemiplegia, showed unadjusted *p*-values below 0.05. To assess the impact of this process on the outcomes of patients with low-risk results, we analyzed the ISI-tested population with Band 1 (‘Sepsis Unlikely’) results, comparing survival in the early (first six months) and late (last six months) implementation periods.

## 3. Results

**Figure 1:**
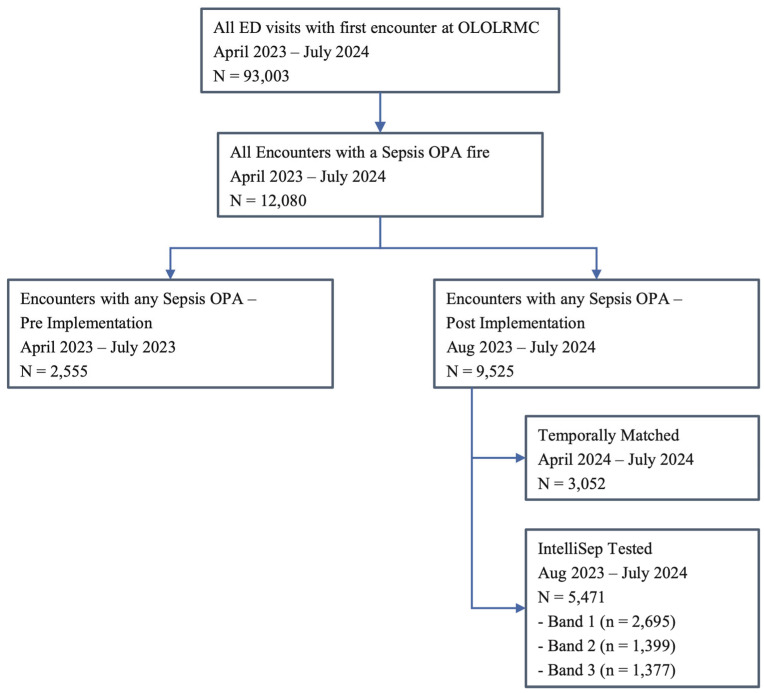
Patient groups for analysis.

Between 23 April 2023 and 31 July 2024, OLOLRMC saw a total of 93,003 ED visits. The pre-implementation period (April–July 2023) included 22,123 ED visits and 2555 OPAs. The post-implementation period (August 2023–July 2024) included 70,880 ED visits and 9525 OPAs. The OPA rate was 11.6% [95%–CI: 11.2–12.1%] during the pre-implementation period, 13.5% [95%–CI: 13.29–13.79%] during the post-implementation period, and 13.0% [95%–CI: 12.6–13.4%] (3052 of 23,490) in the temporally matched cohort (Figure 1).

**Figure 2:**
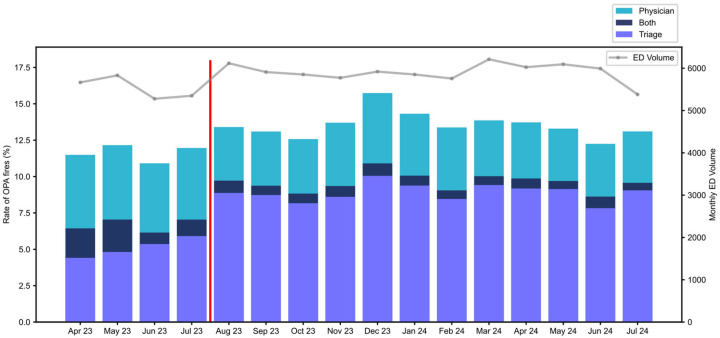
Monthly distribution of our practice advisory (OPA) activations by type: primary/triage, secondary/physician, or both. The overall rate of activations increased slightly over time, driven by an increase in triage activations. The red vertical line marks the implementation date (August 2023).

Patients received both OPAs if they first triggered a triage activation and then a physician activation. Figure 2 shows the monthly distribution of OPA types over the period of analysis. Early in the pre-implementation period (May 2023), both OPAs occurred in a total of 2.2% of total ED patients. To improve the efficiency of operation and minimize the occurrence of both OPA activations, nurse education on the purpose and utility of the ‘Initiate Sepsis Care’ order was provided beginning in June 2023. Thereafter, both activations occurred in 0.5–1.1%.

In August 2023, further nursing education regarding the purpose of identifying suspected infection resulted in an increase in Triage OPAs from 5.9% in July 2023 to 8.9% in August 2023; it remained within the range of 7.8–10.0% thereafter. This education resulted in a small increase in total OPA activation rate. Subsequently, the OPA rates remained stable throughout the post-implementation period. The median time from arrival to Triage OPA activation was 24 min, and 123 min for the provider OPA.

Post implementation, IntelliSep was ordered in 57.5% of patients who triggered any OPA alert, predominantly from the triage OPA (5142 of 5471, 94.80%). The rate of ISI testing increased from 27.7% of the OPA population in August 2023 to a range of 55–67% after September 2023. The provider OPA accounted for the remainder of IntelliSep orders (329 of 5471, 6%). The ordering of IntelliSep outside of either OPA was not available. Among ISI-tested patients, 49.2% resulted in Band 1 (‘Sepsis Unlikely’), 25.5% in Band 2 (‘Sepsis Possible’), and 25.3% in Band 3 (‘Sepsis Probable’).

The average total turnaround time from order to result for the IntelliSep test was 78.2 min, comparable to the turnaround times for lactate (99.3 min) and CBC (72.4 min). The total IntelliSep turnaround time includes the average times from order to collection (39.8 min), collection to laboratory receipt (10.5 min), and laboratory receipt to result reporting (27.9 min).

A similar proportion of patients received a sepsis DRG in the pre-implementation cohort (15.0%, 384 patients), as did the post-implementation cohort (15.3% 1457 patients). Within the IntelliSep-tested population, there were 1218 sepsis DRGs, representing 22.3% of the ISI-tested group. There were 239 sepsis DRGs in the non-ISI-tested population (5.9% of this population). Sepsis diagnosis by DRG increased across interpretation bands: Band 1, 168 of 2961 (5.7%), Band 2, 340 of 1516 (22.4%), and Band 3, 710 of 1495 (47.5%). The rates of sepsis DRGs were not significantly different in the pre-implementation, post-implementation and temporally matched groups (Table 1).

### 3.1. Mortality

**Figure 3:**
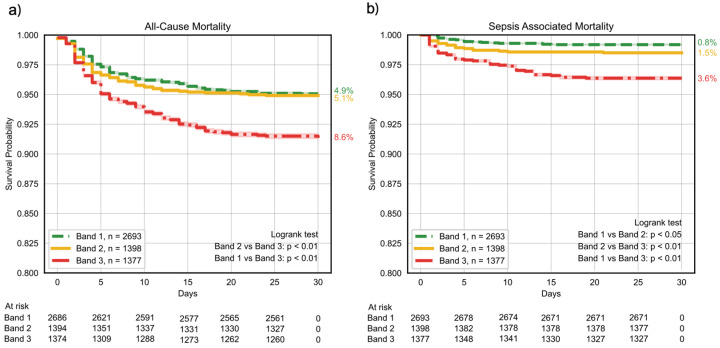
Thirty-day survival probability by the ISI band. (**a**) All-cause in-hospital mortality and (**b**) sepsis-associated mortality post-implementation. Patients receiving a Band 3 ISI score had a significantly higher rate of mortality than those receiving a Band 2 or Band 1 score.

We evaluated the probability of survival to discharge at 30 days of hospitalization, using the endpoints of both mortality from any cause and sepsis-associated mortality (Table 1), stratified by interpretation band. For the endpoint of overall survival (freedom from mortality from any cause), Band 3 had a significantly lower survival than Bands 1 and 2. In patients with a sepsis DRG, the increasing interpretation band was associated with a statistically significant decrease in the probability of survival, from 99.2% in Band 1 to 98.5% in Band 2 (*p* < 0.05 vs. Band 1), and 96.4% in Band 3 (*p* < 0.01 vs. Band 1 and *p* < 0.01 vs. Band 2) (Figure 3).

**Figure 4:**
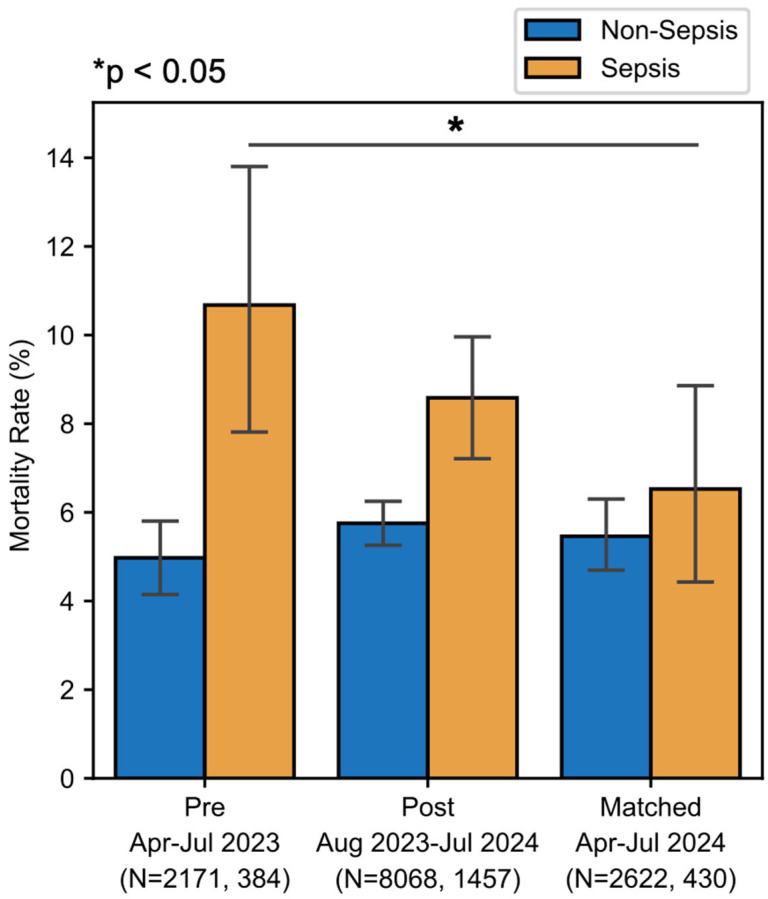
Impact on sepsis-associated mortality. Both the post and matched cohorts showed a decrease in the rate of mortality among sepsis DRGs.

There was a downward trend in sepsis-associated mortality, from 10.9% pre-implementation to 8.6% post-implementation (*p* = 0.203) (Figure 4). When comparing pre-implementation with temporally matched data, there was a statistically significant reduction in sepsis-associated mortality rate from 10.7% in 2023 to 6.5% in 2024 (χ^2^(1, N = 813) = 52.70, *p* < 0.001). This absolute risk reduction of 4.2% in the temporally matched group suggests a number needed to treat of 23.8 patients with a sepsis DRG to prevent one death, and, within this population, a number needed to test of approximately 115 to prevent one death due to sepsis. To ensure that any reduction in sepsis-associated mortality was not due to reclassification, we assessed the mortality of patients with non-sepsis DRGs. There was no significant change in non-sepsis mortality in the total analysis period or in the temporally matched period (Figure 4).

### 3.2. Hospital Legnth of Stay

The implementation of the sepsis intervention targeted improvements in sepsis care, which is reflected in the length of stay for sepsis patients. For patients with a sepsis DRG, there was a statistically significant reduction in HLOS in both the entire implementation period and the temporally matched cohort. Details can be found in Table 2.

### 3.3. Blood Culture Utilization

**Figure 5:**
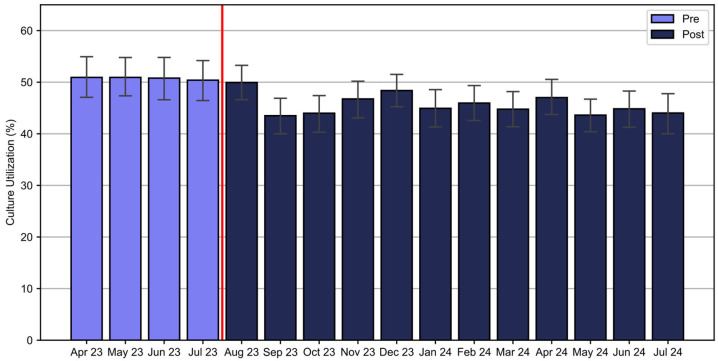
Monthly rate of culture utilization. There was a significant reduction in blood culture utilization in the post-implementation period. The red vertical line marks the implementation date (August 2023).

**Figure 6:**
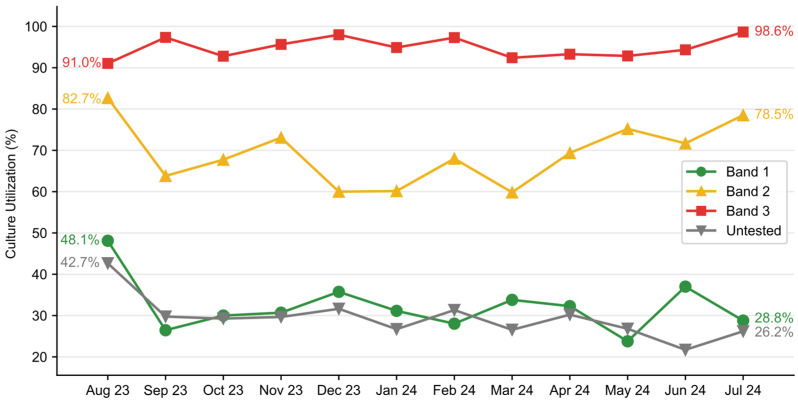
Monthly blood culture utilization by ISI band. Post-implementation trends after August 2023 showed a significant decline in blood culture orders in patients receiving a Band 1 score, while there was a transient decline in blood culture utilization in those receiving a Band 2 score, and an increase in those receiving a Band 3 score.

There was a significant reduction in blood culture utilization from 50.8% of patients receiving cultures in the pre-implementation period to 45.7% in the post-implementation period (χ^2^(1, N = 12,080) = 20.57, *p* < 0.001) (Figure 5). A significant decline in blood culture use was observed in patients with Band 1 results after August 2023, decreasing from 48.1% in August to a range of 23.8–37.0% from September 2023 to July 2024. There was a transient decline in blood culture utilization in Band 2 and an increase in blood culture use in Band 3 patients (Figure 6). Overall, the intervention saved an estimated 944 blood cultures over the one-year post-implementation. Additionally, the rate of contaminated samples decreased from 2.47% in the pre-implementation cohort to 1.97% in the post-implementation cohort and 1.77% in the temporally matched group.

### 3.4. Antimicrobial Utilization

**Figure 7:**
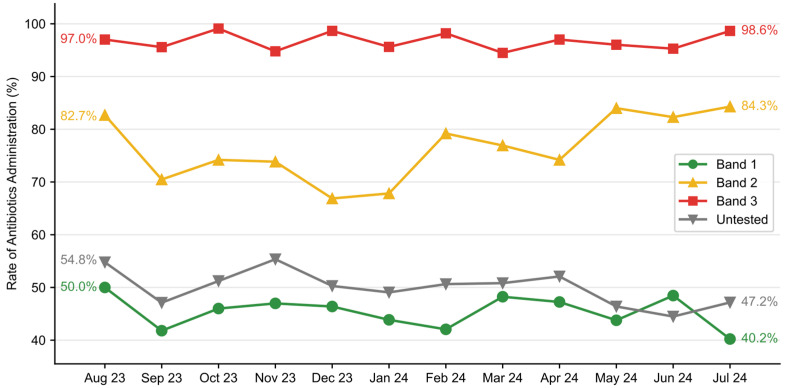
Rate of antibiotic utilization stratified by ISI band. Antibiotic utilization was highest in patients receiving a Band 3 ISI score, slightly lower in those receiving a Band 2 score, and much lower in those receiving a Band 1 score.

Antimicrobial utilization was evaluated as the proportion of patients who received at least one dose of antibiotics in the ED. While overall antimicrobial utilization in the total dataset remained constant (59.3% pre-implementation vs. 59.6% post-implementation), stratification in the rate at which antibiotics were ordered was observed across risk bands in the post-implementation group (Figure 7). Antibiotic utilization was the highest in Band 3 patients, ranging from 94.5% to 99.1% on a monthly basis, slightly lower in Band 2 (66.9–84.3%) and much lower in Band 1 (40.2–50.0%).

## 4. Discussion

This review of the FMOLHS Sepsis Initiative includes the first implementation of IntelliSep into clinical workflow. It shows that standardized screening processes, in combination with protocolized IntelliSep ordering, can yield substantial improvements in outcomes and resource utilization. IntelliSep provides an assessment of the underlying biology of sepsis with results that can support the incorporation of guideline-directed therapy into ED sepsis care.

Expert guidelines recommend active screening procedures to identify patients at risk of sepsis so that they can be identified early and receive timely intervention. Depending on screening thresholds, overtreatment or undertreatment may occur. Overtreatment consumes resources, exposes patients to nonbeneficial therapy, and may lead to diagnostic anchoring which can delay the diagnosis of other life-threatening conditions that resemble sepsis on presentation. On the other hand, undertreatment can result in care delays that adversely impact clinical outcomes. To address these challenges, we employed a two-tiered system with both robust screening and the protocolized ordering of IntelliSep, followed by the implementation of IntelliSep-informed clinical pathways.

To expedite the delivery of care through IntelliSep-informed pathways, we optimized the nurse-driven triage screen. This approach enables the identification of the at-risk population 99 min earlier (median difference) than the EHR-based provider screen. We also optimized the delivery of IntelliSep results by optimizing efficiency in phlebotomy and specimen transport. Consequently, the average total turnaround time for IntelliSep approximates that of a CBC, allowing for the delivery of the result to ED providers in time to influence clinical decision making.

While 13% of all adult ED patients triggered a sepsis OPA in triage, only one in four of these had high-risk IntelliSep results and received care through the ‘Sepsis Probable’ clinical pathway. This pathway encourages the expedited delivery of bundled sepsis care because, based on prior data [16], patients with Band 3 results are at high risk of adverse events. The majority of sepsis DRGs in patients who received IntelliSep testing (58.3%) fell within this group. Furthermore, in our investigation, we found that 27.9% of patients with a sepsis DRG fell within Band 2 and received care via the ‘Sepsis Possible’ pathway.

The addition of IntelliSep appears to have a beneficial impact on sepsis outcomes. When comparing the pre-implementation period to the post-implementation period, we observed a trend toward a reduction in mortality; we noted a statistically significant reduction in sepsis-associated mortality (from 10.7% to 6.5%) in the temporally matched cohort. Based on this analysis of temporally matched data, in the context of our health system, we estimate saving approximately one life per week. In addition to the reduction in sepsis-associated mortality, we also found a decrease in HLOS in patients with a sepsis DRG. The HLOS reduction was significant in both the post-implementation and the temporally matched cohorts.

Blood culture utilization is a vital component of laboratory and diagnostic stewardship [16]. We observed a 10% reduction in the number of blood cultures drawn with improvement in the blood culture contamination rate despite no other PI program targeting blood culture contamination. This reduction was driven by a reduction in the Band 1 population, and it was significant despite a concomitant increase in blood culture utilization in the high-risk population. This finding suggests a reduction in resource utilization and a redistribution of resources, ensuring they are more appropriately allocated to those with the highest probability of benefit. The reallocation of resources was also evident in ED antibiotic utilization. In the post-implementation period, fewer patients in Band 1 received antimicrobials, while more patients in Band 3 received antibiotics, despite no change in overall utilization.

The reduction in and reallocation of resources did not result in adverse outcomes. Moreover, the reduction in sepsis-associated mortality was not associated with an increase in mortality for those without sepsis DRGs. Thus, the reduction in sepsis-associated mortality does not appear to be due to the reclassification of non-survivors into other, non-sepsis DRGs.

Staffing, ED volumes (see Figure 2), and patient characteristics were also unchanged during this time. During the implementation process, we recognized the value of a protocolized approach to change, and the importance of thorough and consistent clinician training. These elements were central to achieving improvements in outcomes and may serve as critical considerations for similar institutions seeking to adopt this model.

There are limitations to the interpretation of these results. First, these findings represent real-world clinical data from a single institution, and, although the patient population is large and diverse, the generalizability to other facilities remains unclear. Additionally, while patient demographics were largely consistent across the pre-and post-implementation periods, and temporal matching likely accounted for variation in disease patterns, some additional time-variant differences in patients were not accounted for.

Data were obtained through an automated query of the EHR, which limited the data that were accessible for analysis. Specifically, the data did not allow for risk-adjustment based on comorbidities. Additionally, analysis of sepsis-associated outcomes relied on sepsis DRGs, rather than formal adjudication, which may introduce a classification bias. Finally, although the implementation of IntelliSep is the major change that differentiates the pre–and post-implementation periods, as with all observational data, we cannot confirm the cause of these findings. Confirmation will require the demonstration of reproducibility at other facilities and/or an interventional study, such as a randomized, controlled trial.

## 5. Conclusions

We showed a process of sepsis care that includes robust screening coupled with IntelliSep, an objective measure of innate immune activity, and that can impact both outcomes and resource use for patients with sepsis as well as those with similarly presenting alternative diagnoses. Successful implementation requires the diligence of the nursing staff, collaboration with the laboratory, commitment from providers, and support from the administration.

## Figures and Tables

**Table 1:** Patient demographics.

Category		Pre-Implementation 4/23–8/23	Post-Implementation 8/23–8/24	Temporally Matched 4/24–8/24
ED Encounters	Total Volume	22,123	70,880	23,490
OPA Alerts	Total	2555	9525	3052
	Triage only	1118 (43.8%)	6272 (65.8%) *	2048 (67.1%) **
	Physician Only	1091 (42.7%)	2770 (29.1%) *	853 (27.9%) **
	Received Both	346 (13.5%)	483 (5.1%) *	151 (4.9%) **
Age	Median (Q1–Q3)	66 (51–76)	66 (53–76)	66 (52–76)
Sex	Female	1220 (47.8%)	4457 (46.8%)	1447 (47.4%) **
	Male	1335 (52.2%)	5048 (53.0%)	1597 (52.3%) **
	Unknown	0 (0.0%)	20 (0.2%)	8 (0.3%) **
Race	Black or African American	1203 (47.1%)	4471 (46.9%)	1422 (46.6%)
	Other/Unknown	105 (4.1%)	438 (4.6%)	149 (4.9%)
	White or Caucasian	1247 (48.8%)	4616 (48.5%)	1481 (48.5%)
Hispanic Ethnicity	No	2494 (97.6%)	9315 (97.8%)	2982 (97.7%)
	Yes	61 (2.4%)	210 (2.2%)	70 (2.3%)
	Unknown	0 (0%)	0 (0%)	0 (0%)
Charlson Comorbidity Index	Index	1.94	1.97	1.98
Sepsis	Has DRG (870–872)	384 (15.0%)	1457 (15.3%)	430 (14.1%)
Discharge to Hospice	No	2440 (95.5%)	8948 (93.9%) *	2859 (93.7%) **
	Yes	115 (4.5%)	573 (6.0%) *	189 (6.2%) **
	Unknown	0 (0.0%)	4 (0.0%) *	4 (0.1%) **
In-Hospital Mortality (30 day)	No	2406 (94.2%)	8932 (93.8%)	2877 (94.3%)
	Yes	149 (5.8%)	589 (6.2%)	171 (5.6%)
	Unknown	0 (0.0%)	4 (0.0%)	4 (0.1%)
Sepsis-Associated Mortality (30 day)	No	2514 (98.4%)	9396 (98.7%)	3020 (99.0%) **
	Yes (of total) [of Sepsis DRG]	41 (1.6%) [10.7%]	125 (1.3%) [8.6%]	28 (0.9%) ** [6.5%]
	Unknown	0 (0.0%)	4 (0.0%)	4 (0.1%) **
Blood Culture Result Rates	True Positive Rate (%)	12.6%	13.0%	14.2%
	Contamination Rate (%)	2.5%	2.0%	1.8%

* Pre-vs.-post (*p* < 0.05), ** Pre-vs.-matched (*p* < 0.05).

**Table 2:** Hospital length of stay (HLOS) for patients with sepsis DRGs before and after the sepsis initiative implementation, compared using the Mann–Whitney U test.

		(N)	Mean HLOS Days	Median HLOS Days	Mann–Whitney U-Test
Sepsis DRGs	Pre-implementation	301	6.72	5	
	All	1171	6.08	5	U = 162,972 *p* = 0.042
	Temporally matched	349	5.96	4	U = 47,834 *p* = 0.048

## Data Availability

The datasets used and/or analyzed during the current study are available from the corresponding author on reasonable request.

## Supplementary Materials

The following supporting information can be downloaded at https://www.mdpi.com/article/10.3390/healthcare13111273/s1, Section S1: Implementation of our lady of the Lake Regional Medical Center Sepsis Learning Initiative; Section S2: Charlson Comorbidity Index comparisons.

## Abbreviations

The following abbreviations are used in this manuscript:

DRG	Diagnosis-related group
ED	Emergency department(s)
EHR	Electronic health record
ISI	IntelliSep Index
OPA	Our practice advisory
PI	Performance improvement
HLOS	Hospital length of stay

## References

[1] Singer M., Deutschman C.S., Seymour C.W., Shankar-Hari M., Annane D., Bauer M., Bellomo R., Bernard G.R., Chiche J.-D., Coopersmith C.M. (2016). The Third International Consensus Definitions for Sepsis and Septic Shock (Sepsis-3). JAMA.

[2] Martin G.S. (2012). Sepsis, severe sepsis and septic shock: Changes in incidence, pathogens and outcomes. Expert Rev. Anti Infect. Ther..

[3] Epstein L., Dantes R., Magill S., Fiore A. (2016). Varying estimates of sepsis mortality using death certificates and administrative codes—United States, 1999–2014. MMWR Morb. Mortal. Wkly. Rep..

[4] Liu V., Escobar G.J., Greene J.D., Soule J., Whippy A., Angus D.C., Iwashyna T.J. (2014). Hospital deaths in patients with sepsis from 2 independent cohorts. JAMA.

[5] Liang L., Moore B., Soni A. (2020). National Inpatient Hospital Costs: The Most Expensive Conditions by Payer, 2017. HCUP Statistical Brief #261.

[6] Newman-Toker D.E., Peterson S.M., Badihian S., Hassoon A., Nassery N., Parizadeh D., Wilson L.M., Jia Y., Omron R., Tharmarajah S. (2022). Diagnostic Errors in the Emergency Department: A Systematic Review.

[7] Rhee C., Kadri S.S., Danner R.L., Suffredini A.F., Massaro A.F., Kitch B.T., Lee G., Klompas M. (2016). Diagnosing sepsis is subjective and highly variable: A survey of intensivists using case vignettes. Crit. Care.

[8] Lopansri B.K., Miller R.R., Burke J.P., Levy M., Opal S., Rothman R.E., D’alessio F.R., Sidhaye V.K., Balk R., Greenberg J.A. (2019). Physician agreement on the diagnosis of sepsis in the intensive care unit: Estimation of concordance and analysis of underlying factors in a multicenter cohort. J. Intensive Care.

[9] Rhee C., Chiotos K., Cosgrove S.E., Heil E.L., Kadri S.S., Kalil A.C., Gilbert D.N., Masur H., Septimus E.J., Sweeney D.A. (2020). Infectious Diseases Society of America Position Paper: Recommended Revisions to the National Severe Sepsis and Septic Shock Early Management Bundle (SEP-1) Sepsis Quality Measure. Clin. Infect. Dis..

[10] Saposnik G., Redelmeier D., Ruff C.C., Tobler P.N. (2016). Cognitive biases associated with medical decisions: A systematic review. BMC Med. Inform. Decis. Mak..

[11] Gossett D.R., Tse H.T.K., Lee S.A., Ying Y., Lindgren A.G., Yang O.O., Rao J., Clark A.T., Di Carlo D. (2012). Hydrodynamic stretching of single cells for large population mechanical phenotyping. Proc. Natl. Acad. Sci. USA.

[12] Crawford K., DeWitt A., Brierre S., Caffery T., Jagneaux T., Thomas C., Macdonald M., Tse H., Shah A., Di Carlo D. (2018). Rapid biophysical analysis of host immune cell variations associated with sepsis. Am. J. Respir. Crit. Care Med..

[13] O’neal H.R.J., Sheybani R., Caffery T.S., Musso M.W., Hamer D., Alwood S.M., Berlinger M.S., Jagneaux T., LaVie K.W., O’neal C.S. (2021). Assessment of a cellular host response test as a sepsis diagnostic for those with suspected infection in the emergency department. Crit. Care Explor..

[14] Agency for Healthcare Research and Quality: About Learning Health Systems. https://www.ahrq.gov/learning-health-systems/about.html.

[15] O’Neal H.R., Sheybani R., Kraus C.K., Self W.H., Shah A.M., Thomas C.B., Tse H.T.K., Scoggins R. (2024). Cellular host response sepsis test for risk stratification of patients in the emergency department: A pooled analysis. Acad. Emerg. Med..

[16] Fabre V., Carroll K.C., Cosgrove S.E. (2022). Blood culture utilization in the hospital setting: A call for diagnostic stewardship. J. Clin. Microbiol..

