# Supplementary material for: Impact of a Sepsis Quality Improvement Initiative on Clinical and Operational Outcomes
Source: Healthcare (Basel). 2025 May 28;13(11):1273. doi: 10.3390/healthcare13111273 (PMC12154479; doi:10.3390/healthcare13111273)
Supplement: Supplementary file 1 [file healthcare-13-01273-s001.zip › healthcare-3605108-supplementary.pdf]

healthcare

MDPI

Supplemental Material

Section S1: Implementation of Our Lady of the Lake Regional Medical Center Sepsis Learning Health Initiative

Brief description of the OLOL Sepsis Learning Health initiative, including screening criteria, OPA's, and treatment pathways.

Our Lady of the Lake Regional Medical Center (OLOLRMC), the flagship hospital of the Franciscan Missionaries of Our Lady Health System, is an 800-bed academic medical center with Level 1 trauma certification in Baton Rouge, Louisiana. In July 2022, FMOLHS developed a Sepsis Learning Health initiative, a sepsis Performance Improvement (PI) program within the health system's Quality and Safety infrastructure. The Agency for Healthcare Research and Quality (AHRQ) defines a learning health system (https://www.ahrq.gov/learning-health-systems/about.html) as one "in which internal data and experience are systematically integrated with external evidence, and that knowledge is put into practice." A major goal of such initiatives is the continual assessment of outcomes to refine process and educate staff, resulting in a feedback loop for continued improvements in care. Thus, a learning health initiative continually analyzes data to result in iterative improvements in process and respective improvement in patient-centered outcomes.

Due to its iterative nature, the FMOLHS Sepsis Learning Health Initiative includes multiple components, implemented over time:

1. Sepsis Clinical Care and Reporting (January 2023): A multidisciplinary team of process engineers, providers, nurses, and IT specialists refine and implement sepsis clinical care and reporting processes.
2. Screening and Diagnostic Processes (April 2023): Screening for patients presenting with signs and symptoms of infection is revised through an Electronic Health Record (EHR; Epic Systems) Our Practice Advisory (OPA; formerly, Best Practice Advisory). This process includes two distinct OPA mechanisms:
    - Primary/Triage Nursing Screen – OPA fires based on logic that uses parameters available at the time of triage. The OPA provides access to a Nurse-Initiated Order Set that includes a preliminary laboratory evaluation consisting of Complete Blood Count (CBC), Comprehensive Metabolic Profile (CMP), and Lactic Acid) for patients presenting with signs and symptoms of infection. Nurses can opt out of the sepsis evaluation in the setting of major trauma or burn. Details of the triage OPA logic are in Figure S1, and a screen shot of the alert is included in Figure S2.
    - Secondary/Provider Screen – OPA fires when Epic Sepsis Model (ESM) v. 1.0 reaches a threshold value of 6 or greater (calculated every 20 minutes). This OPA results in a Provider-Initiated Order for further evaluation of patients with suspicion of sepsis. A screenshot of this alert is included in Figure S3.
3. Regular data analysis (beginning August 2023): Weekly reporting to the implementation team, including compliance with screening mechanisms, resource utilization, and patient-centered outcomes.

 https://doi.org/10.3390/healthcare13111273

Initiate Sepsis Care Order

An 'Initiate Sepsis Care' order is included in the order set attached to both Primary/Triage and Secondary/Provider OPA's. This order activates the sepsis checklist within the EHR, and serves as a flag to indicate that the treatment team has initiated a process to further evaluate for possible sepsis. It does not imply that the patient has sepsis or is actively being treated for it, but that the patient is under investigation. Importantly, if the Initiate Sepsis Care order is placed through the nurse-initiated order set, it suppresses the provider OPA and prevents it from firing, even if the Early Sepsis Model (ESM) reaches the set threshold. Thus, both OPA's activate if the Triage OPA first activated, the nurse did not select the 'Initiate Sepsis Care' order, and the patient subsequently met criteria for the provider OPA.

Triage OPA Criteria & Logic

Criteria

- Vital Signs:
    - Mean Arterial Pressure < 65 mmHg
    - Temperature > 101 F
    - Heart Rate > 100 Beats / Minute
    - Respiratory Rate > 22 Breaths / Minute
- Nurse Evaluation
    - Is a possible infection present?
    - Does the patient have altered mental status?

Logic

The OPA will fire in any of the three following conditions:

1. Mean Arterial Pressure criteria met

2. At least two of Temperature, Heart Rate, and Respiratory Rate met
   and
   Any one of the Nurse Evaluation Questions = Yes

3. Both Nurse Evaluation Questions = Yes

Figure S1: Triage OPA screening criteria and logic.

Implementation of IntelliSep

On August 1, 2023, at OLOLRMC, the IntelliSep test became available for clinical use through the Nurse-initiated order by its inclusion in the triage OPA. On August 23, 2023,

IntelliSep became available for direct ordering by clinicians upon activation of the provider-based OPA.

IntelliSep-Informed Treatment Pathways

Three discrete IntelliSep-informed clinical pathways were developed to help guide ED providers: Band 1 (Sepsis Unlikely), Band 2 (Sepsis Possible), and Band 3 (Sepsis Probable). These pathways are based on the consensus guidelines of the Surviving Sepsis Campaign with specific recommendations informed by multicentered data collected from prior IntelliSep studies.15 These pathways are not protocolized or mandated care, but a guide for ED clinicians to consider in the treatment of individual patients. Details of the pathways can be found in Figures S4–S6.

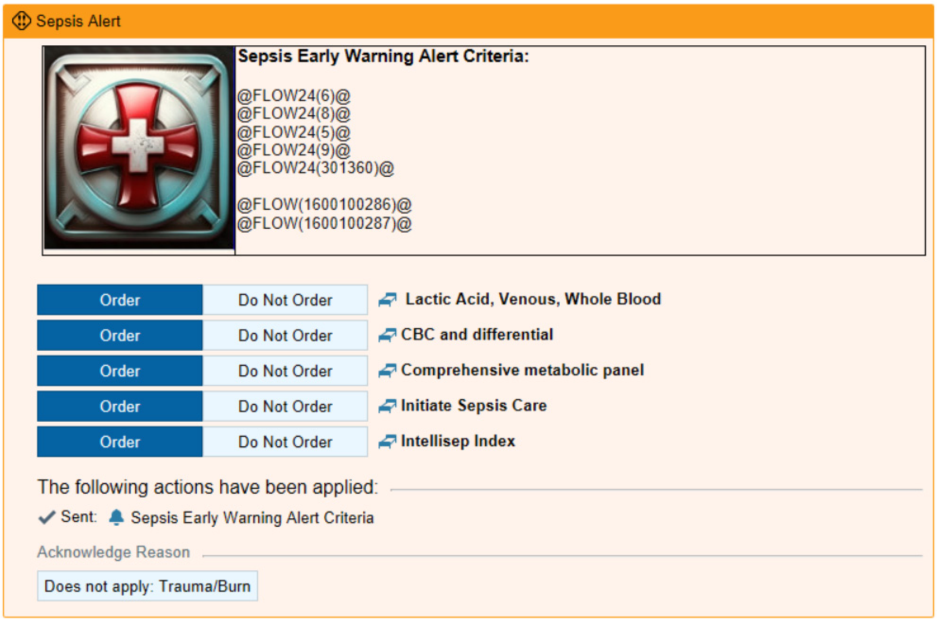

Figure S2: Triage OPA with Associated Nurse-Initiated Order Set

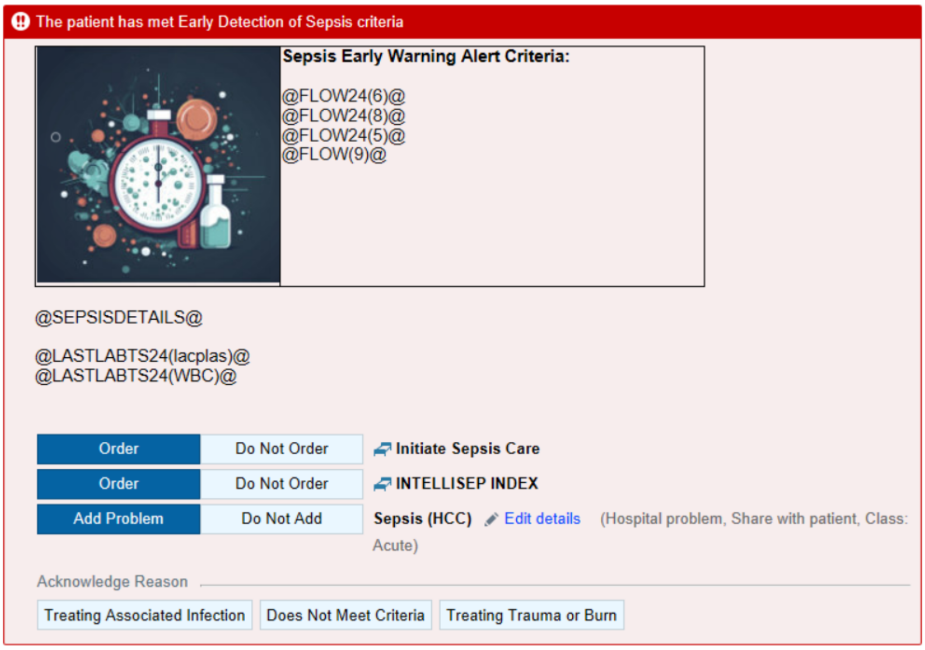

Figure S3: Provider OPA with Associated Order Set

Ultimately, the goal of the IntelliSep-dependent pathways was to inform ED clinicians on four major aspects central to the care of patients who present with signs or symptoms of infection:

1. Risk stratification for adverse outcomes (mortality)
2. Need for pathogen identification (blood cultures)
3. Appropriate antimicrobial utilization
4. Disposition

Providers were formally educated on the IntelliSep-informed treatment pathways through a series of journal clubs and 'lunch-and-learn' activities conducted within the first month of implementation. Following these initial educational activities, no formal education or feedback was provided to clinicians regarding ED interventions.

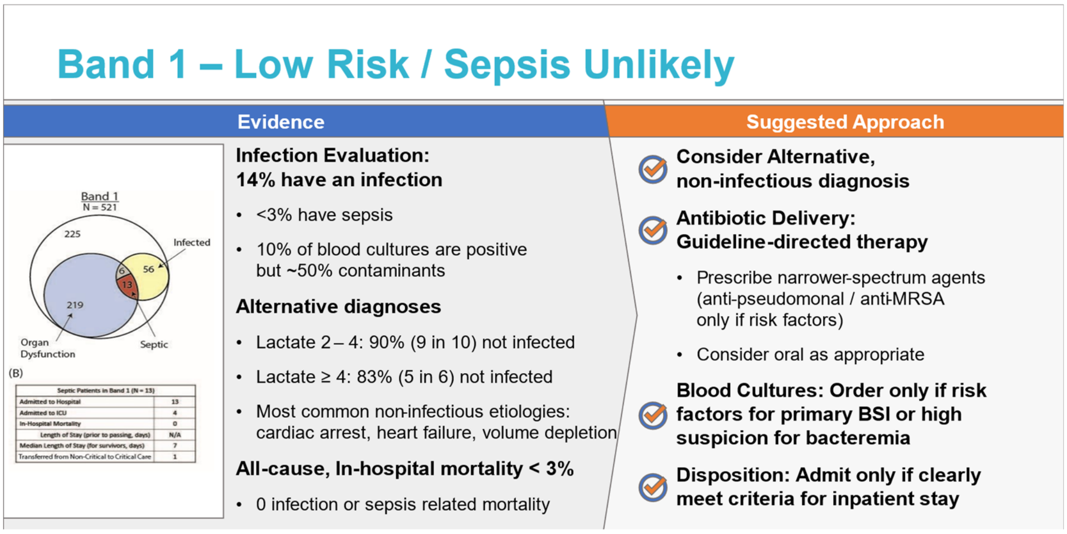

Figure S4: Band 1 (Sepsis Unlikely) pathway

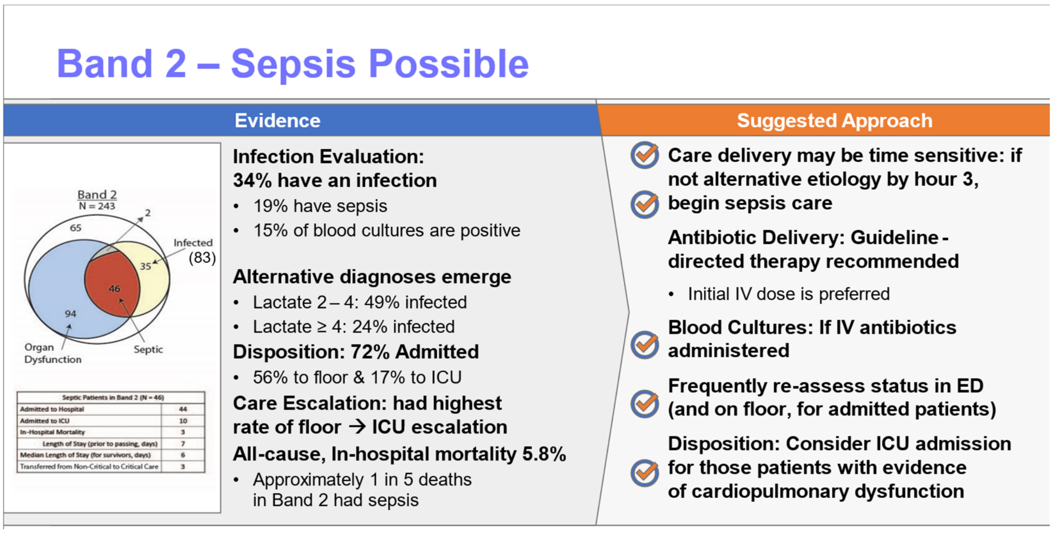

Figure S5: Band 2 (Sepsis Possible) pathway

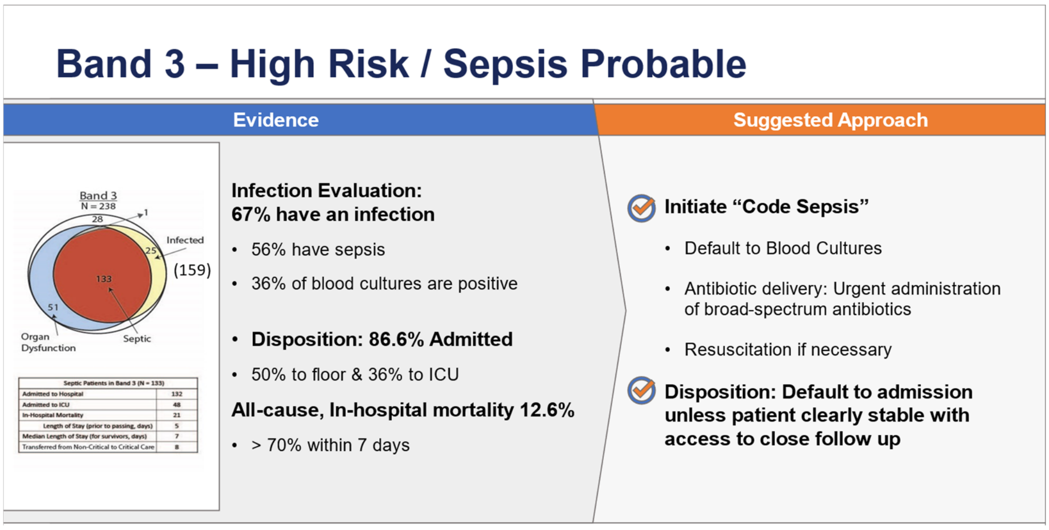

Figure S6: Band 3 (Sepsis Probable) pathway

Section S2: Charlson Comorbidity Index Comparisons

Table S1. Charlson Comorbidity Category Comparisons Across Groups

	Counts			Percentage (%)			P-Values (Chi-square test of proportions)		Chi-squared Values		Corrected P-Values (Benjamini/ Hochberg correction)	
**Charlson Category**	**Pre**	**Post**	**Matched**	**Pre**	**Post**	**Matched**	**Pre vs Post**	**Pre vs Matched**	**Pre vs Post**	**Pre vs Matched**	**Pre vs Post**	**Pre vs Matched**
AIDS/HIV	47	179	60	1.8	1.9	2	0.895	0.730	0.017	0.119	0.951	0.776
Cancer	251	969	327	9.8	10.2	10.7	0.603	0.275	0.271	1.193	0.854	0.519
Cerebrovascular Disease	261	1128	359	10.2	11.8	11.8	0.022*	0.066	5.242	3.386	0.188	0.343
Chronic Pulmonary Disease	472	1830	558	18.5	19.2	18.3	0.398	0.854	0.713	0.034	0.697	0.854
Congestive Heart Failure	758	2702	855	29.7	28.4	28	0.197	0.173	1.665	1.854	0.669	0.421
Connective Tissue Disease-Rheumatic Disease	65	232	84	2.5	2.4	2.8	0.754	0.629	0.099	0.233	0.915	0.766
Dementia	161	680	221	6.3	7.1	7.2	0.140	0.164	2.183	1.935	0.593	0.421
Diabetes with complications	625	2229	708	24.5	23.4	23.2	0.263	0.268	1.255	1.226	0.697	0.519
Diabetes without complications	730	2623	812	28.6	27.5	26.6	0.300	0.101	1.073	2.696	0.697	0.343
Metastatic Carcinoma	94	352	125	3.7	3.7	4.1	0.969	0.423	0.002	0.643	0.969	0.653
Mild Liver Disease	161	640	202	6.3	6.7	6.6	0.451	0.631	0.568	0.231	0.697	0.766
Moderate or Severe Liver Disease	43	121	33	1.7	1.3	1.1	0.110	0.052	2.561	3.766	0.593	0.343
Myocardial Infarction	188	747	261	7.4	7.8	8.6	0.416	0.101	0.662	2.690	0.697	0.343
Paraplegia and Hemiplegia	83	405	142	3.2	4.3	4.7	0.022*	0.008*	5.233	7.119	0.188	0.130
Peptic Ulcer Disease	33	144	46	1.3	1.5	1.5	0.411	0.495	0.677	0.465	0.697	0.701
Peripheral Vascular Disease	288	1062	353	11.3	11.1	11.6	0.862	0.730	0.030	0.119	0.951	0.776
Renal Disease	709	2684	883	27.7	28.2	28.9	0.668	0.328	0.184	0.956	0.874	0.558

p = NS unless otherwise indicated; * Denotes $p < 0.05$

Table S2. Charlson Comorbidity Index Composite Results

	Pre	Post	Matched
count	2555	9525	3052
mean	2.71	2.72	2.77
std	2.49	2.53	2.60
min	0	0	0
25%	1	1	1
50%	2	2	2
75%	4	4	5
max	14	16	16

Pre vs Post: $p = 0.862575$

Pre vs Matched: $p = 0.695394$

Table S3. Age-Adjusted Charlson Comorbidity Index Composite Results

	Pre	Post	Matched
count	2555	9525	3052
mean	4.73	4.78	4.80
std	3.25	3.25	3.33
min	0	0	0
25%	2	2	2
50%	5	5	5
75%	7	7	7
max	16	18	17

Pre vs Post: $p = 0.460898$

Pre vs Matched: $p = 0.80927$
